# Renal Recovery following Liposomal Amphotericin B-Induced Nephrotoxicity

**DOI:** 10.1155/2019/8629891

**Published:** 2019-01-28

**Authors:** Heather A. Personett, Bryce M. Kayhart, Erin F. Barreto, Pritish Tosh, Ross Dierkhising, Kristin Mara, Nelson Leung

**Affiliations:** ^1^Department of Pharmacy, Mayo Clinic, Rochester, MN, USA; ^2^Department of Pharmacy, Robert D. and Patricia E. Kern Center for the Science of Health Care Delivery, Mayo Clinic, Rochester, MN, USA; ^3^Division of Infectious Diseases, Mayo Clinic, Rochester, MN, USA; ^4^Division of Biomedical Statistics and Informatics, Mayo Clinic, Rochester, MN, USA; ^5^Division of Nephrology and Hypertension, Mayo Clinic, Rochester, MN, USA

## Abstract

**Background:**

Acute kidney injury (AKI) is a common complication of treatment with liposomal amphotericin B (LAmB). The trajectory of renal recovery after LAmB-associated AKI has not been well described, nor has effect of LAmB dose on recovery of renal function been explored.

**Objective:**

Characterize the pattern of renal recovery after incident AKI during LAmB and determine potential influencing factors.

**Methods:**

This retrospective cohort study analyzed patients who developed a ≥50% increase in serum creatinine while on LAmB. Patients were followed up until complete renal recovery or death or for 30 days, whichever occurred first. The primary outcome was complete renal recovery, defined as serum creatinine convalescence to within 10% of the patient's pretreatment baseline. Multivariable modeling was used to identify independent predictors of renal recovery.

**Results:**

Ninety-eight patients experienced nephrotoxicity during LAmB, 94% of which received doses <7 mg/kg/day. Fifty-one patients at least partially recovered renal function and, of these, 32 exhibited complete recovery after a mean 9.8 ± 7.8 days. No statistical relationship was found between LAmB dose at the time of AKI or cumulative exposure to LAmB and the likelihood of renal recovery. Concomitant nephrotoxins, age, and pretreatment renal function did not modify this effect in multivariable analysis.

**Conclusion and Relevance:**

Our data suggests that LAmB dose did not impact the likelihood of renal recovery. Additional investigation is needed to confirm these findings when aggressive dosing strategies are employe. Additional research is also warranted to further characterize the course of recovery after LAmB-associated nephrotoxicity and comprehensive spectrum of renal outcomes.

## 1. Introduction

Amphotericin B is a broad spectrum antifungal agent with over half a century of use in the treatment of invasive fungal infections [1]. Liposomal amphotericin B (LAmB), though less toxic than other formulations, is nonetheless still associated with a high frequency of adverse effects. These include infusion reactions, hepatotoxicity, and, perhaps most troublesome, nephrotoxicity [2].

Based on the available literature, doubling of SCr during treatment with LAmB occurs in 7.6%-19% of patients who receive doses of 5 mg/kg/day or less [3–6]. Unfortunately, treating life-threatening mold infections sometimes necessitates aggressive escalation of amphotericin B doses beyond this threshold for successful pathogen eradication [7, 8]. At higher LAmB doses, nephrotoxicity reportedly increases, befalling up to 43% of individuals exposed to 15 mg/kg/day [9].

Though the relationship between LAmB and kidney injury has previously been described, little is known about the course of renal recovery after LAmB-induced nephrotoxicity. The timeframe and likelihood of complete renal recovery are unknown, as is the influence of escalating doses on the probability of nephrotoxicity reversal. Historical data links amphotericin B deoxycholate exposure with permanent kidney injury [10], though current practice has shifted toward increased use of the newer lipid-based formulations. These are suggested to have distinct mechanisms of nephrotoxicity; thus extrapolation of this data to patients receiving LAmB is likely inappropriate [11]. More recent retrospective analyses describe the economic burden and mortality associated with nephrotoxicity from amphotericin B nephrotoxicity but do not report on the outcome of renal recovery [12, 13].

The marked differences in reported renal outcomes throughout the literature along with increased utilization of lipid formulations of amphotericin B, and at higher doses, highlight the need for further investigation of nephrotoxic events after LAmB exposure. The purpose of this study was to characterize the pattern of renal recovery after incident AKI during LAmB and determine potential influencing factors, particularly those related to dose.

## 2. Methods

### 2.1. Study Design and Participants

This retrospective cohort study included hospitalized adults (>18 years of age) at Mayo Clinic in Rochester, Minnesota, who received intravenous LAmB between January 2008 and March 2015. The study protocol was approved by the Institutional Review Board and the need for informed consent was waived. Patients were identified using an institution-specific antimicrobial administration record and were included if AKI developed during LAmB therapy, at least 24 hours after the index administration. Excluded patients had end-stage renal disease, underwent renal replacement therapy in the seven days prior to the development of nephrotoxicity, or did not consent to have their medical records used for research. In an effort to omit AKI cases primarily due to causes other than LAmB, we also excluded patients who exhibited a rise in SCr exceeding 0.3 mg/dL within 24 hours of the first LAmB dose or who were exposed to intravenous contrast within 48 hours of AKI [14]. While possible, onset of injury this early after drug exposure would be unlikely explained by a single dose of the medication, but rather alternate causes.

At Mayo Clinic Hospital, Rochester, LAmB is the preferred and default amphotericin B product. There is no specific dosing algorithm or dose escalation protocol in use at the institution. Actual body weight is used for dose calculation except in patients weighing >120 kg or with a BMI >40 kg/m^2^, where adjusted body weight is used. Salt loading with pre- and post-LAmB dose infusions of 250 mL 0.9% sodium chloride was standard practice as a toxicity-prevention strategy [15], though this is not required. There is no predefined dose-reduction protocol in the event of a nephrotoxic event; the events are handled on a case-by-case basis.

### 2.2. Definitions

Acute kidney injury was defined as an increase of at least 50% in SCr from the pretreatment value and the date of AKI was considered to be day 0 in the analysis. This definition has been used frequently in the previously published literature for amphotericin B and was chosen for consistency [9, 12, 13]. Urine output was not included in the AKI definition as this was inconsistently documented in hospital ward patients. The degree of renal injury was also described using the SCr component of Acute Kidney Injury Network criteria for staging [16]. Pretreatment SCr was defined as the measurement drawn within 24 hours of LAmB initiation. Pretreatment creatinine, rather than baseline creatinine, was chosen as the index marker of renal function to minimize the confounding effects of events occurring prior to the initiation of LAmB. Baseline creatinine was also collected for descriptive purposes and was defined as the lowest value documented in the six months prior to LAmB-associated AKI, or a value designated as the patient's individual baseline by a nephrologist. Complete recovery of AKI was defined as a return to within 10% of pretreatment SCr (pretreatment SCr value +10%) within the first 30 days after AKI [17]. A patient was considered to have a partial recovery if the SCr returned to within 11-25% of the pretreatment value (pretreatment SCr value +11-25%) by the end of follow-up (Supplementary Appendix Figure S1).

### 2.3. Follow-Up and Endpoints

The primary outcome was complete recovery of kidney injury within the first 30 days after nephrotoxicity. Secondary outcome measures included partial renal recovery, mean extent of GFR recovery, and freedom from renal replacement therapy, if applicable. Patients were followed until complete recovery, death, or discharge, or for 30 days after LAmB-associated nephrotoxicity, whichever occurred first. At the end of follow-up, the time to complete recovery and partial recovery was recorded. The time to any recovery was defined as the time to the first detected partial or complete recovery. If the first detected recovery was a complete recovery, a partial recovery was assumed to have occurred on the same day. Daily SCr values were captured when available. For the multivariable analysis, exposure to nephrotoxins occurring during LAmB therapy was collected, including vancomycin, aminoglycosides, polymyxins, angiotensin converting enzyme inhibitors, nonsteroidal anti-inflammatory agents, calcineurin inhibitors, methotrexate, platinum-based antineoplastics, foscarnet, and cidofovir. Additionally, comorbidities that could compromise an individual's likelihood of renal recovery were gathered if present, including cardio- or hepatorenal syndrome, septic shock, or hypotension requiring vasopressor support.

### 2.4. Statistical Analysis

Baseline patient characteristics were described with frequencies and percentages for categorical variables and means ± standard deviations (SD) or medians and interquartile ranges (IQR) for continuous data. Time to reversal of nephrotoxicity was described using Kaplan-Meier curves. A multivariable Cox proportional hazard model was used to estimate the effect of cumulative LAmB dose on kidney injury recovery, after adjusting for a prespecified set of covariates including age, concomitant nephrotoxins, and baseline renal function [12, 18]. Cumulative LAmB dose was treated as a time-dependent covariate. A p value < 0.05 was considered statistically significant.

## 3. Results

### 3.1. Baseline Patient Characteristics

**Figure 1:**
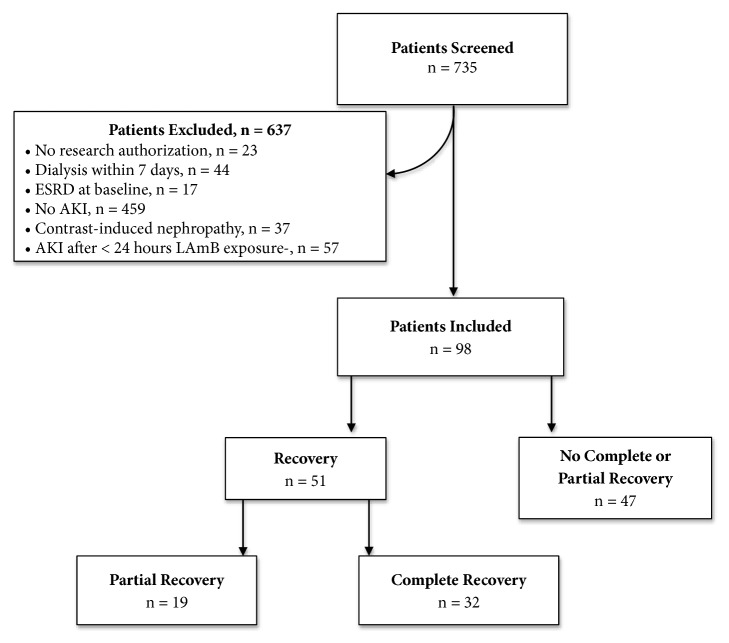
Consort diagram: 735 patients who received LAmB at Mayo Clinic between 2008 and 2015 were screened. 637 were excluded, leaving 98 patients available for analysis.

A total of 735 unique patients with any exposure to LAmB were screened and 98 included after application of eligibility criteria. The most common reason for exclusion was no nephrotoxicity during LAmB therapy (N = 459; 62% of patients screened) (Figure 1). For the 89 patients in whom a baseline SCr was available, the mean ± SD value was 0.8 ± 0.2 mg/dL. The mean pretreatment SCr was 0.9 ± 0.3 mg/dL (p = 0.012 for the different from baseline) which resulted in a mean estimated glomerular filtration rate (eGFR), calculated with the CKD-EPI equation [18], of 91.6 ± 21.2 mL/min/1.73m^2^. In 17 (17%) patients, LAmB therapy was initiated in the intensive care unit. Vancomycin was the most frequently encountered concomitant nephrotoxin, used in 50% of patients, followed by calcineurin inhibitors in 17 (17%), trimethoprim-sulfamethoxazole in 10 (10%) and angiotensin converting enzyme inhibitors in 10 (10%) patients. Additional baseline characteristics are displayed in Table 1.

### 3.2. Acute Kidney Injury

After initiation of treatment with LAmB, the median time to AKI was 3.6 days (IQR 2.3 – 7.5). The majority of AKI cases, 48 (48%), were AKIN stage I with 33 (33%) and 18 (18%) cases of stages II and III AKI, respectively. The average LAmB dose at the time of AKI was 4.6 mg/kg/day, and 92 (94%) patients received a LAmB dose less than 7 mg/kg/day. Thirty (31%) patients received a cumulative dose greater than 5 grams. Eight patients (8%) required renal replacement therapy for management of their kidney injury. Median time to initiation of renal replacement therapy was 4.5 days (range 2-17). Fifty patients (51%) were concomitantly receiving at least one other nephrotoxin at the time of AKI, most often vancomycin (Table 2).

### 3.3. Outcomes

In 43 patients (44%), LAmB was discontinued within 24 hours of AKI onset. Fifty patients (51%) had LAmB discontinued beyond 24 hours. There was no statistically significant difference in absolute SCr increase between those where LAmB was stopped within 24 hours and those where the drug was continued (0.1 ±0.1 versus 0.07 ±0.1, p=0.31). There was also no statistically significant difference in the percent (%) SCr increase between those where LAmB was stopped within 24 hours and those where the drug was continued (14.7 ±19.1 versus 10.0 ±14.6, p=0.36). Five patients remained on LAmB for at least 30 days, 3 of whom exhibited no recovery of renal function. We did not find an association between early discontinuation of LAmB (i.e., within 24 hours) and renal recovery (HR 0.9, 95% CI 0.5–1.6, p=0.82). In fact, no association was found between discontinuing LAmB at any time and recovery (HR 1.2, 95% CI 0.6–2.4, p=0.55). In the patients who continued LAmB therapy despite AKI, dose reductions were infrequent, occurring in 15 (27%) patients overall.

**Figure 2:**
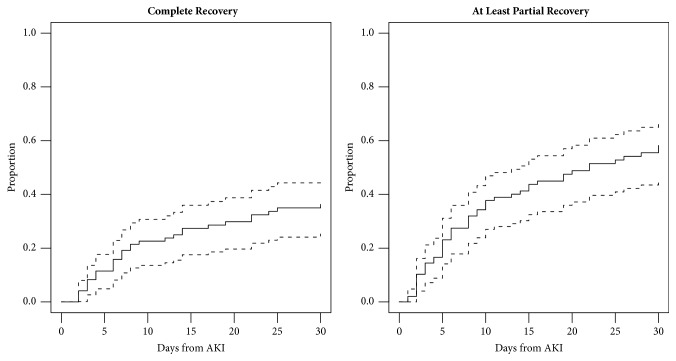
Kaplan Meier curves depicting complete recovery and at least partial recovery over the course of 30 days from AKI. The dotted lines represent 95% confidence intervals.

Complete recovery of renal function occurred in 32 patients (33%) after a mean ± SD of 9.8 ± 7.8 days since AKI onset (Figure 2). Any recovery (partial or complete) occurred in 51 patients (51%), leaving 47 patients (49%) in whom SCr failed to recover to within 25% of their pretreatment value by the end of follow-up. Of the patients exhibiting recovery, the median time to partial recovery was 6 days (IQR 3–15). In those without complete or partial recovery, the mean relative eGFR loss from pretreatment eGFR was 45.7% at last follow-up. Eight patients required dialysis and, of these, four died. Of the remaining four, two were free from renal replacement therapy by the end of the follow-up period. Of the two who remained on dialysis at 30 days, one continued to receive LAmB. In total, nine (9%) patients died and none exhibited renal recovery prior to their death.

There was no statistically significant difference in the rates of complete (unadjusted HR 2.7, 95% CI 0.4–19.7, p=0.33) or partial recovery (unadjusted HR 4.9, 95% CI 0.7–35.8, p=0.12) in patients receiving LAmB doses <7 mg/kg/day compared with those who received >7 mg/kg/day. Additionally, cumulative LAmB dose at any point during therapy, number of concurrent nephrotoxins, baseline renal function, and age did not impact recovery of any type on univariable analysis (Table 2). Cumulative LAmB dose was not found to be associated with renal recovery after adjusting for covariates including age, baseline GFR, and nephrotoxin exposure (Table 3). Episodes of shock, cardiorenal syndrome, and hepatorenal syndrome were infrequent and, thus, were not included in the multivariable analysis.

## 4. Discussion

In this retrospective study of 98 individuals with LAmB-associated nephrotoxicity, only 36% of the cohort exhibited complete renal recovery within 30 days. Moreover, approximately half failed to experience SCr recovery to within 25% of their pretreatment value during the follow-up period. Likelihood of renal recovery was not predicted by daily dose at the time of AKI, cumulative LAmB dose at the time of AKI, number of concomitant nephrotoxin exposures during the treatment course, comorbidities, baseline renal function, or age.

Amphotericin B is thought to have several mechanisms by which it precipitates nephrotoxicity, including arteriolar vasoconstriction and direct tubular injury [19]. It would be expected that the course of recovery would differ between these two phenotypes. To complicate matters further, most research characterizing amphotericin B-induced renal injury is derived from patients who received amphotericin B deoxycholate. Deoxycholate, a bile salt derivative used to solubilize amphotericin B, has been shown to be nephrotoxic even in the absence of amphotericin B [11, 20]. The relatively rapid course of at least partial recovery suggests that many of these patients who recovered may have had a prerenal mechanism of injury, though not directly studied in this work.

Luber and colleagues summarized 178 cases of amphotericin B exposure, of which 8–50% experienced a nephrotoxic event [18]. The authors state that there were no cases of irreversible nephrotoxicity. This starkly contrasts with our data in which almost half of the cohort did not return to within 25% of their pretreatment renal function. Unfortunately, Luber and colleagues did not report the duration of follow-up, nor the amphotericin B formulation used in their study population, making direct comparisons difficult.

Interestingly, in the present study, the dose of LAmB was not correlated with an individual's likelihood of recovering from his or her nephrotoxic event in our study. This was true for the cumulative LAmB dose prior to AKI, the daily dose at the time of AKI, and LAmB exposure after AKI. Although high rates of nephrotoxicity have been reported in patients who receive a cumulative total dose of 5 grams or more of amphotericin B deoxycholate [13], we are not aware of any published data that has established a reliable cumulative dose threshold associated with irreversible renal injury for liposomal amphotericin B. Though one-third of the cohort received cumulative doses greater than 5 grams and average cumulative doses were 2.4 grams at the time of nephrotoxicity and 4.9 grams overall, our findings indicate that the likelihood of renal recovery at 30 days was unrelated to dose and instead is perhaps attributable to other, as yet unidentified, factors.

Though 51% of the cohort was exposed to concomitant nephrotoxins in addition to LAmB, we found no association between renal recovery and concomitant nephrotoxin use. It has previously been reported that male sex, higher weight, and concomitant use of cyclosporine, vancomycin, and angiotensin converting enzyme inhibitors are all independently associated with a higher risk of LAmB-associated nephrotoxicity [12, 18, 21]. We elucidated no relationship between these factors and an individual's likelihood of recovering from a nephrotoxic event secondary to LAmB exposure.

Definitions of renal recovery after AKI are evolving and no specific one has been used consistently in the context of LAmB-associated nephrotoxicity [22]. In fact, the majority of existing literature does not elaborate on the employed definition of recovery, making a direct comparison impossible. The definition used in the present study has been employed previously, primarily in research pertaining to renal recovery after the use of continuous renal replacement therapy [23–25]. Return to within 10% or 25% of pre-LAmB SCr may be a more strict definition thus decreasing the incidence of complete or partial recovery as defined by this study relative to previous work. The Acute Disease Quality Initiative consensus statement describes a poorer prognosis for patients whose renal injury fails to rapidly improve and suggests assessing renal recovery at 90 days following AKI to determine if chronic renal injury has resulted [26]. Unfortunately, due to limited follow-up data available at 90 days, this was not feasible in the present study.

Our study is not without limitations. First, the issue of prescriber bias is inherent in an analysis of this nature. Physicians may be more predisposed to treat sicker patients with higher doses of LAmB due to a perceived risk of treatment failure. As such, we collected data regarding additional exposures that could indicate a higher severity of illness, such as requirement for vasopressor support, presence of hypoperfusing states such as hepato- or cardiorenal syndrome, and the patients' location during treatment (i.e., ICU or general ward). These events occurred infrequently and thus were not included in the multivariable modeling, potentially limiting the applicability of these findings to the most critically ill patients. We also captured exposure to nephrotoxic medications commonly used in this population. While unlikely, we cannot rule out that administration of other rarely utilized nephrotoxins may have contributed to study findings. Additionally, adherence to salt loading was not explicitly collected therefore we cannot confirm the magnitude of influence, if any, of this practice on the outcome of renal recovery. The definition of AKI in the current investigation was chosen based on those used in prior studies of LAmB nephrotoxicity in an attempt to enhance generalizability of the data. This lacks the sensitivity of AKIN or other definitions which include urine output as a criterion and may have resulted in an underestimation of the rate of AKI, particularly that which was AKIN stage I. Also, it is possible that full recovery of LAmB-associated nephrotoxicity may take longer than 30 days in some patients and their recovery would not have been captured in this analysis. This time frame was chosen based on widely available follow-up data and adds value to existing literature by providing the first defined length of follow-up, furthering our understanding of the clinical course of LAmB-associated nephrotoxicity.

Despite the limitations mentioned above, this is, to our knowledge, the largest investigation of the reversibility of LAmB-associated nephrotoxicity performed to date. More efforts should be made to describe the course of renal recovery in patients with LAmB-associated AKI and the factors which influence it.

## 5. Conclusion

Our data suggests that neither LAmB dose at the time of AKI nor cumulative exposure to LAmB impact the likelihood of renal recovery. Further investigation is needed to confirm these findings when aggressive dosing strategies are utilized. Additional research is also warranted to further characterize the course of recovery after LAmB-associated nephrotoxicity, including the comprehensive spectrum of renal recovery and long-term renal outcomes.

## Figures and Tables

**Table 1:** Baseline characteristics.

**Characteristic**	**Included Patients** **N = 98**
**Underlying Disease**	
** Hematological malignancy, n (**%**)**	53 (54)
** Allogeneic stem cell transplantation, n (**%**)**	14 (14)
** Autologous stem cell transplantation, n (**%**)**	4 (4)
** Solid Organ Transplant, n (**%**)**	9 (9)
** Heart, n (**%**)**	5 (5)
** Kidney, n (**%**)**	1 (1)
** Liver, n (**%**)**	2 (2)
** Lung, n (**%**)**	1 (1)
** Other, n (**%**)**	37 (37)
**Baseline Serum Creatinine, mg/dL**	
Mean (SD)	0.8 (0.2)
Median (IQR)	0.7 (0.6-0.8)
**Pretreatment creatinine, mg/dL**	
Mean (SD)	0.9 (0.3)
Median (IQR)	0.8 (0.7-1.0)
Mean Estimated GFR^b^ (SD), mL/min/1.73m^2^	91.6 (21.2)

^a^ Body mass index.

^b^ Glomerular filtration rate, calculated by CKD-EPI.

**Table 2:** Univariate Cox models.

**Characteristic **	**Summary Data**	**HR for At Least Partial Recovery**	**p-value**	**HR for Complete Recovery**	**p-value**
**Age, mean (SD)**	56 (14.6)	0.99 (0.81, 1.20)^a^	0.88	0.88 (0.69, 1.13)	0.32
**Sex**					
Male, n (%)	63 (64.3)	0.92 (0.52, 1.63)	0.78	0.84 (0.41, 1.70)	0.62
Female, n (%)	35 (35.7)	1.00			
**Race**					
Caucasian, n (%)	87 (35.7)	0.70 (0.30, 1.63)	0.40	0.56 (0.22, 1.46)	0.24
Other, n (%)	11 (11.2)	1.00			
**BMI per kg/m** ^**2**^ **, mean (SD)**	28.5 (7)	1.03 (0.98, 1.07)	0.23	1.01 (0.96, 1.06)	0.82
**Pretreatment serum creatinine per mg/dL, mean (SD)**	0.9 (0.3)	1.10 (0.33, 3.73)	0.88	1.27 (0.27, 6.03)	0.76
**Renal replacement therapy requirement**					
Yes, n (%)	8 (8.2)	0.47 (0.11, 1.94)	0.30	5.48 (0.32, 93.61)	0.24
No, n (%)	90 (91.8)	1.00			
**Daily LAmB dose in mg at time of AKI (per 100 mg), mean (SD)**	288.5 (244)	0.97 (0.85, 1.10)	0.59	0.94 (0.80, 1.03)	0.43
**Pre-AKI cumulative LAmB dose (per 5,000 mg), mean (SD)**	2445.2 (3144.1)	0.87 (0.51, 1.48)	0.60	0.87 (0.46, 1.63)	0.66
**Cumulative LAmB dose** **∗** ^**b**^ ** (per 5,000 mg), mean (SD)**	4985.8 (6659.8)	0.84 (0.58, 1.21)	0.36	0.81 (0.50, 1.31)	0.39
**Concomitant Vancomycin** **∗**					
Yes, n (%)	32 (32.7)	0.79 (0.19, 3.28)	0.74	1.50 (0.35, 6.48)	0.59
No, n (%)	66 (67.3)	1.00			
**Total Concomitant Nephrotoxins** **∗** ** (per 1 nephrotoxin)**		1.36 (0.82, 2.25)	0.24	1.49 (0.81, 2.71)	0.20

*∗* indicates time-dependent variables.

^a^HR per decade increase.

^b^ LAmB prior to AKI+ LAmB after AKI (time dependent).

**Table 3:** Multivariable Cox model.

**Variable**	**Hazard Ratio for At Least Partial Recovery** ** (95**%** CI)**	**p-value**
**Cumulative LAmB dose** ^a^ ** (per 5,000 mg)**	0.78 (0.52, 1.17)	0.23
**Concomitant nephrotoxins at AKI (per 1 nephrotoxin)**	0.81 (0.51, 1.27)	0.35
**Concomitant nephrotoxins after AKI (per 1 nephrotoxin)**	1.59 (0.88, 2.87)	0.12
**Age (per decade)**	1.03 (0.82, 1.29)	0.82
**Baseline eGFR** ^b^ ** (per 5 mL/min/1.73m** ^**2**^ **)**	1.00 (0.93, 1.09)	0.91

^a^ LAmB prior to AKI+ LAmB after AKI (time dependent).

^b^ Glomerular filtration rate, calculated by CKD-EPI.

## Data Availability

The data used to support the findings of this study are included within the article.

## Supplementary Materials

Supplementary Materials
** Supplementary Appendix. Supplemental Figure S1.** Flowchart visualizing enrollment, dose-group assignment, and follow-up. In this example, the patient would be included in the high dose group because the LAmB dose at the time of AKI was > 7 mg/kg/day. They would then be followed until complete recovery, death, or discharge, or for 30 days after LAmB-associated nephrotoxicity, whichever occurred first. Outcomes were subdivided according to study definitions into no recovery, partial recovery, and complete recovery.
