# Supplementary material for: Renal Recovery following Liposomal Amphotericin B-Induced Nephrotoxicity
Source: Int J Nephrol. 2019 Jan 28;2019:8629891. doi: 10.1155/2019/8629891 (PMC6369474; doi:10.1155/2019/8629891)

Supplementary Appendix

Supplemental Figure S1. Flowchart visualizing enrollment, dose-group assignment, and follow-up. In this example, the patient would be included in the high dose group because the LAmB dose at the time of AKI was > 7 mg/kg/day. They would then be followed until complete recovery, death, discharge, or for 30 days after LAmB-associated nephrotoxicity, whichever occurred first. Outcomes were subdivided according to study definitions into no recovery, partial recovery, and complete recovery.

Figure S1 – Sample patient enrollment schematic

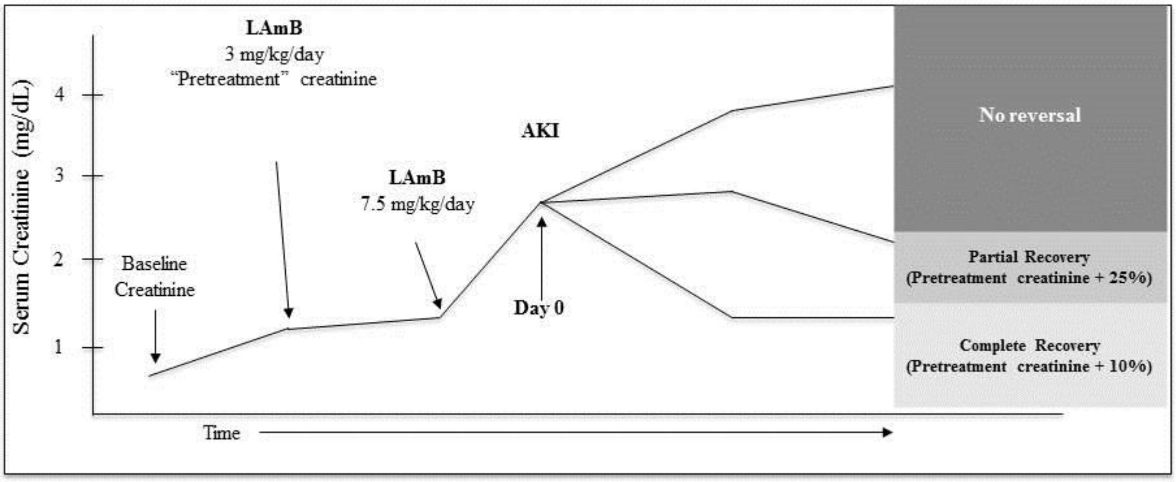

Supplement: Supplementary Materials — Supplementary Appendix. Supplemental Figure S1. Flowchart visualizing enrollment, dose-group assignment, and follow-up. In this example, the patient would be included in the high dose group because the LAmB dose at the time of AKI was > 7 mg/kg/day. They would then be followed until complete recovery, death, or discharge, or for 30 days after LAmB-associated nephrotoxicity, whichever occurred first. Outcomes were subdivided according to study definitions into no recovery, partial recovery, and complete recovery. [file 8629891.f1.pdf]
